# Does active participation in organised physical activity contribute to children’s achievement of the 24-hour movement guidelines? findings from the family physical activity, sedentary behaviour, and sleep (FAMIPASS) study

**DOI:** 10.1186/s12889-025-23136-x

**Published:** 2025-05-26

**Authors:** Erik Sigmund, Dagmar Sigmundová, Jaroslava Voráčová, Michal Vorlíček, Jan Dygrýn

**Affiliations:** 1 Faculty of Physical Culture, Institute of Active Lifestyle, Palacký University Olomouc, Olomouc, Czechia Czechia; 2 Faculty of Physical Culture, Department of Social Sciences in Kinanthropology, Palacký University Olomouc, Olomouc, Czechia Czechia

**Keywords:** Organised physical activity, 24-hour movement behaviour, Mother, Father, Accelerometer

## Abstract

**Background:**

The development of child’s lifestyle occurs within regular 24-hour movement patterns under the guidance of parents. Accelerometer-based monitoring allows for the capture of these 24-hour movement patterns of behaviour. Therefore, the aim of this study was to reveal whether active participation in organised physical activity (OPA) contributed to the achievement of the World Health Organization’s (WHO) 24-hour movement behaviour guidelines (24-hMBGS) among 3–10-year-old children, considering the influence of parents’ movement behaviour and families’ material background.

**Methods:**

The 24-hour movement behaviour (24-hMB) of 348 child-parent pairs (with at least one parent) was continuously monitored for 7 days via ActiGraph accelerometers placed on the non-dominant wrist. Children’s adherence to the WHO’s 24-hMBGS was analysed using logistic regression analysis. The socioeconomic status (SES) of families was measured using the Family Affluence Scale. Body mass level was determined according to body mass index gender- and age-specific WHO reference data. Univariate analysis of variance/Pearson’s chi-square test was used to test differences in sedentary behaviour duration/excess body weight between active participants and non-participants in OPA.

**Results:**

Active 3–10-year-old participants in OPA, compared to non-participants, were significantly more likely to meet at least two of the 24-hMBGS (77.7% vs. 66.4%, *p* = 0.008), had significantly shorter daily sedentary time (by 30 min per day, *p* = 0.001), and a significantly lower prevalence of excessive body weight (10.26% vs. 24.87% *p* < 0.001). Engaging in OPA significantly (*p* < 0.01) helped 3–10-year-old children achieve at least two of the 24-hMBGS, regardless of their gender, age, excess body weight, or family SES. In addition, a mother’s non-excessive body weight and achievement of at least two of the WHO’s 24-hMBGS significantly (*p* < 0.05) contributed to children attaining at least two of the WHO’s 24-hMBGS.

**Conclusions:**

Even in young children, active participation in OPA tend to contribute to a healthier lifestyle profile, characterised by shorter sedentary behaviour and lower excess body weight, with a significant influence from the mother’s movement behaviour.

## Introduction

Human behaviour unfolds in a 24-hour rhythm, with the repetition of the main components of movement behaviour - sleep, sedentary behaviour (SB), and physical activity (PA). Adherence to the recommended combination of these components is associated with desirable health benefits in children and young people [1]. Shaping the composition of children’s movement behaviour and lifestyle takes place primarily under the guidance of parents, who are considered the guardians of their children’s health-related movement behaviour [2]. This includes providing emotional support, modelling PA, promoting shared PA, setting rules to limit SB and screen time (ST), establishing consistent bedtime routines, and maintaining device-free bedrooms [2–4].

Although PA represents the shortest of the 24-hour movement behaviour (24-hMB) components, it appears that increasing PA to at least brisk walking intensity, with the inclusion of high-intensity exercise, has the strongest positive association with metabolic health in children than simply reducing SB [5]. Children aged 7–10 years who regularly participate in OPA team sports - soccer and handball) are significantly more likely to meet the recommendations for moderate to vigorous PA (MVPA) than their peers who do not participate in OPA [6]. In addition, regular (twice per week for 45 min) and long-term (≥ 30 months) participation in organised sports (soccer and swimming) by 3–5-year-old children results in significant improvements in gross motor development, fundamental and specific physical and sport skills [7]. Moreover, 6-year-olds and older children who participate in regular organised sports are more likely to be confident and demonstrate higher degrees of assertiveness, as well as higher degrees of academic and social competence than children who do not participate in any OPA [8]. For preschool children, however, the potential impact of OPA on child health indicators and adherence to the 24-hMBGS remains understudied. This is despite the fact that early childhood has been identified as a critical period for instilling and developing healthy behaviours, such as being physically active [9, 10]. Active participation of young children in OPA positively influences social skills, pro-social behaviours, and self-regulation [9]. Additionally, adherence to at least two of the three 24-hMBGS is positively associated with improved mental health, as well as overall and physical development in pre-schoolers [10, 11]. However, the results are still inconsistent and inconclusive, and further research with stronger methodological design and rigor is needed [1, 9, 10, 12].

The FAMIly Physical Activity, Sedentary behaviour and Sleep (FAMIPASS) study [13] was initiated as a scientific response to the alarmingly increasing prevalence of obesity and the decreasing prevalence of completing 60 min of MVPA per day in 11–15-year-old Czech adolescents, especially in younger age groups (11 years) and among adolescents with low socioeconomic status (SES) [14, 15]. The FAMIPASS study is a nationally representative longitudinal study investigating parent-child relationships in accelerometer measured 24-hMB and lifestyle related indicators (such as body weight levels, leisure-time activity, diet/bedtime/ST rules and routines), considering the SES background of families with preschool children. Previous FAMIPASS studies have indicated the significant role of maternal PA/sleep behaviour in the adherence of their 3–10-year-old offspring to PA/sleep guidelines [16, 17], the absence of ST devices in the bedroom, and reduced sedentary behaviour to achieve sleep recommendations [17]. In addition, significant associations have been found between MVPA and total PA in parent-child pairs across all gender combinations [16]. Moreover, it has been found that 3–10-year-old overweight children exhibited significantly lower PA and higher SB than their non-overweight peers [18]. However, no significant differences were found in the prevalence of excess body weight in children with respect to children’s age, gender, family SES or maternal and paternal obesity [18]. A rigorous analysis of 3–10-year-old Czech children’s adherence to 24-hMBGS, including the factor of active participation in OPA, is still lacking. Therefore, the main aim of this study was to reveal whether active participation in OPA helps 3–10-year-old children meet the WHO’s 24-hMBGS, and whether this achievement is moderated by parental movement behaviour and families’ material background.

## Methods

### Design and setting

The FAMIPASS study is a nationally representative longitudinal study investigating parent-child relationships in accelerometer measured 24-hMB and lifestyle related indicators to find and describe healthy patterns of behaviour to prevent the onset/development of excess body weight in young children [13, 16, 18]. Participating families were recruited through stratified sampling of kindergartens and primary schools from rural and urban areas of Bohemia, Moravia and Silesia [13]. Data collection took place between March 2022 and May 2023 during regular kindergarten/school terms, excluding multi-day holidays and public holidays. Inclusion criteria for families in the longitudinal study were: (a) having at least one child aged 3–10 years, (b) absence of illness/limitations not allowing them to follow the daily kindergarten/school and family-related routine, and (c) willingness to participate voluntarily in the study [13]. The present study elaborates and presents the results of the first wave of FAMIPASS data collection (a one-time cross-sectional study).

### Participants

Of the 860 (100%) families contacted, 552 (64.2%) provided written informed consent to participate in the study and 502 (58.4%) of them began continuous seven-day 24-hMB monitoring. A total of 472 (54.9%) families completed full seven-day monitoring, and 348 (40.5%) families (including at least one parent-child pair) with valid data on 24-hMB, OPA participation, and family background were included in the final data set for statistical analyses. Reasons for excluding 124 parent-child pairs from the final data set were as follows: insufficient valid accelerometer data-defined as fewer than three valid school days and one weekend day (*n* = 65); missing anthropometric measures, parental education level, or family background information necessary to determine the SES of families (*n* = 26); and missing OPA data (*n* = 33). The final dataset contained valid and complete data on 24-hMB, OPA participation, and anthropometric and sociodemographic characteristics of 348 children (50.3% girls) and their parents (Table 1).

**Table 1:** Basic characteristics of participating girls and boys, their parents, and the socioeconomic status of their families

Characteristics Participants in organised PA	GIRLS (*n* = 175) (*n* = 73; 41.71%)	BOYS (*n* = 173) (*n* = 84; 48.55%)
*Age (months, M ± SD)	77.07 ± 20.23	78.03 ± 19.40
*Height (cm, M ± SD)	120.02 ± 12.34	120.74 ± 11.57
*Weight (kg, M ± SD)	23.15 ± 6.71	23.26 ± 5.90
**Body Mass Index** (kg·m⁻², M ± SD)	15.84 ± 2.62	15.78 ± 2.25
**Excess body weight**	17.24%	19.30%
^**#**^ **Socioeconomic status**		
Low	13.6%	12.0%
Middle	66.3%	70.7%
High	20.1%	17.3%
*Mother/father age (years, M ± SD)	36.85 ± 4.47/40.23 ± 5.63	36.88 ± 4.70/39.32 ± 4.96
*Maternal/paternal university education	55.83%/42.28%	59.01%/38.58%
*Maternal/paternal obesity	11.18%/14.71%	9.28%/12.41%

Note: n– number; PA– physical activity; cm– centimetre; kg– kilogram; m– metre; M– mean; SD– standard deviation; *obtained directly from the responses in the family diary; ^#^obtained from calculations based on the Family Affluence Scale-III items

### Measurement of 24-hMB and lifestyle indicators

24-hMB monitoring was performed using ActiGraph accelerometers (wGT3X-BT for children and GT9X Link for parents; ActiGraph LLC, Pensacola, FL, USA) placed on the wrist of the non-dominant hand in both parents and children. During a joint informational meeting at the kindergarten/school, the researchers instructed parents on how to attach the accelerometers to their wrists and record time for themselves and their children in a family diary several times a day. The diary, designed in a spreadsheet format, allowed parents to easily record daily activities such as wake-up time, arrival at kindergarten/school, physical education classes, leaving kindergarten/school, OPA units (practices, coach/leader-led lessons), and bedtime. The 24-MB monitoring began at midnight on the day of the parent-researcher meeting.

Parental and child accelerometers were individually initialised using ActiLife software version 6.13.4 (ActiGraph LLC, Pensacola, FL, USA) for each family member separately based on the information provided in the written informed consent. Accelerometers recorded triaxial acceleration data at a sampling rate of 100 Hz. All accelerometer data sets were analysed using the R GGIR version 2.7-1 package and previously established cut-offs for participants’ 24-hMB intensity levels. Specifically, SB was defined as acceleration values less than 36 milligravity units (m*g*); light PA as 36–200 m*g*; MVPA as 201–706 m*g*; and vigorous PA as values equal to or greater than 707 m*g* [19, 20]. The default settings for non-wear time detection in Part 1 of the R package GGIR were used. Specifically, the algorithm requires that the standard deviation of a rolling signal window be close to the sensor’s noise level. When this condition is met, GGIR labels the middle 15 min of that 60‐minute window as non‐wear [19]. Sleep time, defined as the time from lying in bed to waking, was determined using the default setting of a heuristic algorithm analysing the distribution of angle changes [20]. As a condition for including accelerometer data in the final dataset, at least three kindergarten/school days and one weekend day had to be observed for at least 16 h per day, and accelerometer data had to be available for each 15-min interval of the 24-h cycle [20]. Average daily sleep time, SB, light PA, MVPA, vigorous PA, and total PA were calculated as the weighted arithmetic mean of these activities performed during the kindergarten/school and weekend days (weighted mean=[(average day×5)+(average weekend×2)]/7).

### Measuring anthropometric characteristics, determining families’ socioeconomic status and assessing children’s leisure screen time

The family diary contained graphic instructions with written descriptions for measuring participants’ height and weight at home. Height and weight measurements were taken in the morning, after waking up, in their underwear, and before breakfast. Parents measured height and weight to the nearest 0.5 cm/0.1 kg, respectively [21]. Parental measurement of children’s height and weight at home has been confirmed to be sufficiently valid for the calculation of body mass index (BMI) and subsequent identification of overweight and obesity in 4-10-year-old children [22, 23].

An estimate of family SES was provided by the Family Affluence Scale-III (FAS), which was part of the family diary. The FAS comprised several simple-to-answer questions created to quantify material assets in the families with children [24]. The content of the six FAS questions and their answer options were as follows: having one’s own bedroom for each child in the family (0 or 1); number of computers in the household (0, 1, 2 and ≥ 3); number of cars owned for family use (0, 1 and ≥ 2); number of foreign holidays taken in the past year (0, 1, 2 and ≥ 3); ownership of a dishwasher (0 or 1); number of bathrooms in the household (0, 1, 2 and ≥ 3). The sum of all six questions produced a summary score from which three categories of family SES were calculated as follows: the lowest/highest 20% of the summary score characterised low/high SES families, while the range of 21–79% characterised medium SES families [16]. FAS summary score correlated with parent reported income with Eta-squared close to 0.30. The pooled test-retest reliability correlation was *r* = 0.90 [16]. In the socioeconomic conditions of Czechia, the FAS was validated in relation to the disposable household income (Pearson correlation *r* = 0.77 *p* < 0.001 [25].

The family diary included the following two questions on children’s leisure ST, which were answered by parents: ‘How many hours a day do you usually spend in your leisure time on weekdays/weekends watching TV, DVDs, videos (including YouTube or similar online services)?’ and ‘How many hours a day do you usually spend in your leisure time on weekdays/weekends playing games on a computer, games console (PlayStation, Xbox etc.), smartphone, tablet or similar electronic device?’ Questions were categorised by weekdays and weekends. There were nine different answers for each question (none, half an hour, 1, 2, 3, 4, 5, 6 and 7 or more hours per day). Reliability and validity of the 7-day recall questions were demonstrated in comparison with the 7-day 24-hour diaries for weekdays and weekends [26]. Total ST was calculated as the sum of the weighted arithmetic means of weekday and weekend ST (weighted mean=[(average weekday×5)+(average weekend×2)]/7).

### Classification of participants according to 24-hMBGS

Preschool children aged 3–4 years with a total PA of at least 180 min/day, MVPA of at least 60 min/day, ST limited to a maximum of 1 h/day, and a daily sleep duration of 10–13 h were assessed as meeting the 24-hMBGS [27]. Children aged ≥ 5 years who cumulatively performed 60 min of MVPA per day, had no more than 2 h/day of ST, and slept 9–11 h/day were classified as meeting the 24-hMBGS [28, 29]. Adult parents aged 18–64 years whose SB did not exceed 8 h/day, who performed at least 150 min/week of MVPA and slept 7–9 h/day were assessed as meeting the 24-hMBGS [30].

### Ethics

This study is part of a longitudinal project whose baseline data collection, methodology and the follow-up research phase were approved by the institutional Ethics Committee for Research of the Faculty of Physical Culture, Palacký University Olomouc (baseline: protocol code 25/2021 on 28 February 2021 and follow-up: protocol code 102/2023 on 10 November 2023). The consent/permission protocols of the Ethics Committee included the conditions of the research implementation - anonymous written informed consent for parents and their minor offspring and data protection rules. The accelerometers used were disinfected and sealed in an anonymous envelope with a family code along with a family diary before the research. Participation in the research was voluntary and free of charge. No participant was penalised in any way for interrupting or not completing the research, not returning the family diary, or damaging or losing the accelerometer. Participating families received individualised feedback on the results of their own 24-hMB in the form of printed colour graphic sheets with explanatory comments. Schools received a thank-you letter and a certificate of participation in the FAMIPASS study.

### Data management and statistical analysis

**Fig. 1:**
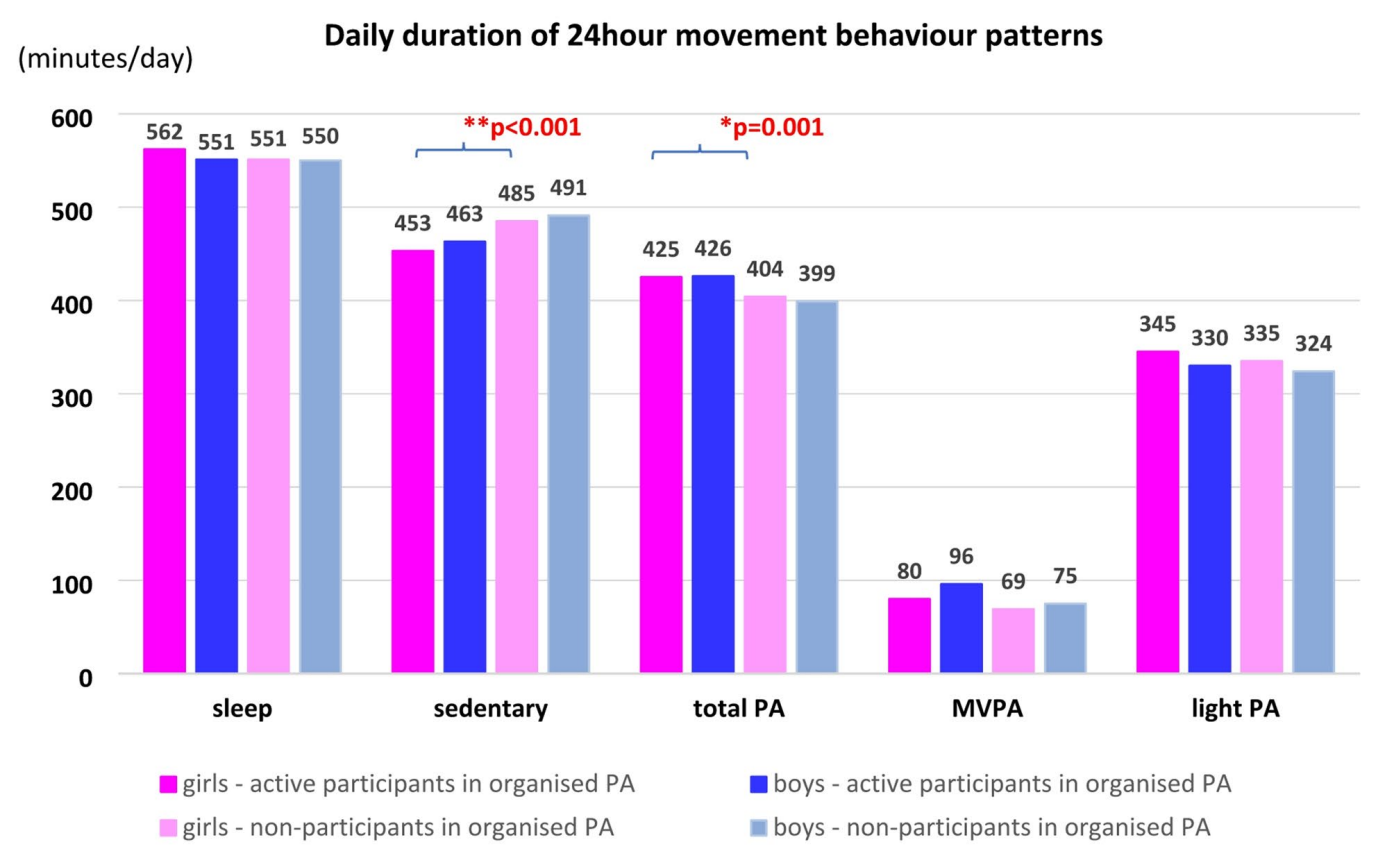
Comparison of 24-hour movement behaviour patterns between non-participants and active participants in organised physical activity units (PA– physical activity; MVPA– moderate-to-vigorous physical activity)

**Fig. 2:**
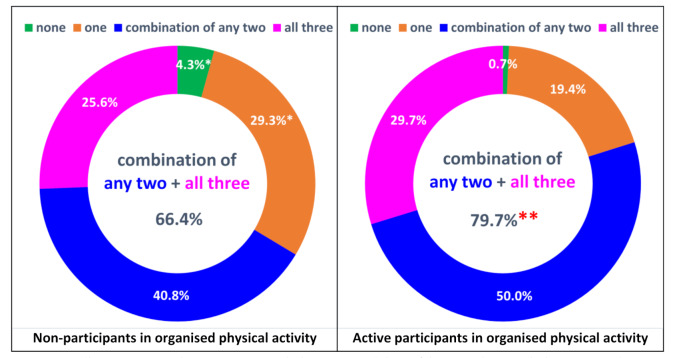
Adherence to 24-hour movement behaviour guidelines (sleep, sedentary behaviour, and physical activity) by non-participants and active participants in organised physical activity units (*/** significant at the 0.05/<0.01 p-level (Pearson's chi-square test)

After applying the inclusion criteria, all data processing and statistical analyses were performed in the Statistical Package for the Social Sciences for Windows V.26 software environment (IBM Corp, Armonk, NY, USA). In the final dataset, all accelerometer-based (PA, SB and sleep) and family diary-based (anthropometric characteristics, ST, SES, OPA) variables were checked for the presence of apparent confounders and outliers. Participants’ BMI was calculated as the ratio of body weight (kg) to the square of body height (m^2^). Subsequently, BMI z-scores were calculated for children based on gender- and age-specific WHO reference data [31]. Excess body weight in children was represented by BMI z-scores > 1 standard deviation (SD) [31–33], whereas in adults, it was defined as a BMI ≥ 25 kg/m^2^ [34]. Basic descriptive characteristics for anthropometric, parental, and family-related variables of girls and boys are presented as arithmetic means and SDs or percentages (%) (Table 1). The Pearson Chi-square test (χ^2^) was used to test differences in the percentage of excess body weight, participation in OPA, or SES between girls and boys. The Kolmogorov-Smirnov test confirmed the normal distribution of the variables: time spent in SB, total PA, MVPA, light PA and sleep. Daily duration of 24-hMB patterns (i.e., sleep, SB, total PA, MVPA, light PA) is presented as arithmetic means (Fig. 1). Differences in 24-hMB patterns between groups of children with active participation in OPA and non-participants in OPA were tested by Univariate analysis of variance. Adherence to 24-hMBGS is presented as percentages separately for active participants in OPA and non-OPA (Fig. 2). Associations between child and parent characteristics and children’s adherence to at least two of the three 24-hMBGS were revealed through logistic regression analysis in the mother-child and father-child models (Table 2). In the regression analyses, one parent was represented multiple times in families with multiple children in the household that participated in the study (20.6% of the final participating families had more than one child included in the analyses). Results of logistic regression analyses were expressed using odds ratios (ORs) and 95% confidence intervals (95% CIs). The alpha significance level was set at a minimum of 0.05.

## Results

No statistically significant differences were found between girls and boys regarding excess body weight (χ^2^ = 0.017, *p* = 0.897), gender-related participation in OPA (χ^2^ = 1.644, *p* = 0.200), or family SES (χ^2^ = 1.977, *p* = 0.372); therefore, subsequent statistical analyses between active participants and non-participants in OPA could have been performed cumulatively for both girls and boys, as well as across different SES categories of families (Table 1).

In summary, girls and boys with active OPA participation showed, on average, 24 min per day significantly (F = 11.27, *p* = 0.001) higher total PA and 30 min per day significantly (F = 14.03, *p* < 0.001) lower SB than children without OPA participation. The difference of 24 min in total PA between active participants in OPA and non-participants in OPA was due to a significant difference in MVPA (17 min, F = 21.53, *p* < 0.001) and a non-significant difference of 8 min in light PA (Fig. 1). In addition, children with active participation in OPA also showed, on average, almost 6 min less ST (117.11 vs. 122.82 min per day) than children without participation in OPA. The average duration of OPA for active participants was 87.64 min (79.31 min in preschool children aged 3-5.9 years and 89.78 min in children aged 6–10 years) per monitored week, implemented in two sessions. Children with active participation in OPA had a significantly lower prevalence of excess body weight than children without participation in OPA (10.26% vs. 24.87% χ^2^ = 12.23, *p* < 0.001).

Day-to-day differences in the duration of each component of 24-hMB were associated with adherence to the 24-hMBGS. Non-participants in OPA were significantly more likely to comply with none of the guidelines or only one recommendation in isolation, compared to active participants in OPA (Fig. 2). Conversely, active participants in OPA were significantly (*p* = 0.008) more likely to meet any two and all three 24-hMBGS than non-participants in OPA (79.7% vs. 66.4%) (Fig. 2).

For child-related variables, only active participation in OPA was significantly related to the likelihood of meeting the 24-hMBGS. Children with active participation in OPA had significantly higher odds of meeting at least two of the 24-hMBGS than children without participation in OPA, regardless of gender, age (3-5.9 vs. 6–10 years), and body weight level (Table 2). Mothers had a stronger influence than fathers on their children’s adherence to the 24-hMBGS. A mother’s non-excessive body weight and her own attainment of at least two of the three 24-hMBGS significantly helped children meet at least two of the 24-hMBGS, regardless of parental educational level or SES (Table 2).

**Table 2:** Association of family-related variables to children’s chances of achieving any combination of at least two or more WHO guidelines for 24-hour movement behaviour

Family-related variables		Achieving any combination of at least two or more WHO guidelines for 24-hour movement behaviour			
		**mother-child** model (268 mother-child pairs)		**father-child** model (207 father-child pairs)	
		OR	95% CI	OR	95% CI
**Child**					
Gender	boy	Ref.		Ref.	
	girl	0.933	0.517–1.681	0.998	0.514–1.939
Age category	3-5.9 years	Ref.		Ref.	
	6–10 years	0.585	0.307–1.116	0.608	0.296–1.247
Excessive body weight	no	Ref.		Ref.	
	yes	2.091	0.851–5.138	1.033	0.408–2.615
Organised PA	no	Ref.		Ref.	
	yes	2.967**	1.542–5.707	2.816**	1.385–5.727
**Parent**					
University education	no	Ref.		Ref.	
	yes	1.302	0.717–2.361	0.691	0.346–1.379
Socioeconomic status	low	Ref.		Ref.	
	middle	0.400	0.147–1.085	0.881	0.283–2.737
	high	0.888	0.261–3.021	1.524	0.397–5.854
Excessive body weight	no	Ref.		Ref.	
	yes	0.505*	0.266–0.958	0.541	0.263–1.112
Achieving ≥ 2 of the	no	Ref.		Ref.	
WHO’s 24-hMBG	yes	2.525**	1.307–4.878	1.291	0.662–2.519

Note: PA– physical activity; OR– odds ratio; CI– confidence interval; Ref.– reference group; */**significant at the 0.05/0.01 p-level

## Discussion

Previous FAMIPASS-related studies have presented the research methodology [13], parent-child sleep patterns and adherence to sleep-only recommendations [17], and the parent-child relationship in total PA and MVPA [16]. However, the factors of children’s participation in OPA and their adherence to 24-hMBGS remained unresolved. Despite this, more than 40% of girls (41.7%) and nearly half of all boys (48.6%) participated in at least two OPA sessions per week during the study.

Thus, monitored Czech 3–10-year-olds adhered to all three 24-hMBGS at higher rates compared to the results of large systematic reviews and meta-analyses, which found compliance rates of 11.3% in 3–5-year-old pre-schoolers, 10.3% in 6–12-year-old schoolchildren [35], and 14.4%/11.9% in 3–5-year-old pre-schoolers from high-income/upper-middle-income countries [36]. In contrast to our findings, more recent studies using accelerometers to monitor PA and sleep, and determining ST by parental proxy report, also revealed lower adherence rates to the 24-hMBGS (11.3% [37]; 7% [38–40]; 2.9% [41]).

The higher rates of adherence to the 24-hMBGS among Czech children, compared to data from other studies, may be explained by the spring and autumn monitoring periods, which featured favourable weather conditions that allowed for outdoor leisure time. Another factor that contributed to the higher adherence of Czech preschool children to 24-hMBGS compared to their foreign peers can be attributed to the compulsory daily kindergarten program, which included supportive elements to routine PA (e.g., daily outdoor walks, active games both outside and inside), interruptions to long-term SB (e.g., group meal preparation, dressing), and adherence to afternoon sleep. In the first and second year of primary school, the curriculum includes regular physical education classes (twice a week), active time during breaks and an afternoon free club with the possibility of implementing PA of the teacher’s choice. Completion of daily lessons in the preschool/school was the criterion for inclusion of the child in the final sample in this study [18]. A similar adherence rate of 23.6% to the three 24-hMBGS was found in a study of Finnish 3–6-year-old pre-schoolers who also attended daytime early education [42]. A very high adherence rate of 43% to all three 24-hMBGS was found in a Canadian study of 3–5-year-old children [11], but in a relatively small sample of participants (*n* = 95) who were also recruited from a sports-based program. Similar to the Canadian children, it was found that Czech children with active participation in OPA most often met a combination of at least two of the three 24-hMBGS.

Surprisingly, active participation in OPA was the only one of the observed child-related variables (gender, age category, body weight level, OPA) that significantly increased the odds of children meeting any combination of at least two of the 24-hMBGS in both the mother-child and father-child regression models. Although OPA occupied only a small proportion of the minimum 4-day 24-hMB monitoring (2.75% in pre-schoolers aged 3–6 years; 3.11% in school-aged children), it appears that active participation in OPA can “harmonize” a child’s daily routine toward the recommended sleep duration, sedentary time, and PA, regardless of the child’s gender, age, or body weight level. These findings are consistent with the results of the ToyBox-Study, which showed that participation in sports clubs and team sports significantly helped pre-schoolers [43] achieve all three 24-hMBGS. However, in contrast to Belgian pre-schoolers from the ToyBox-Study, university education of fathers was not associated with higher odds of achieving the 24-hMBGS in Czech 3–10-year-old children [43].

The mother’s adherence to at least two of the three WHO’s 24-hMBGS and her normal body weight were significantly associated with higher odds of child achieving any combination of at least two or more 24-hMBGS, whereas neither the father’s variables nor the family’s SES showed such an association. This finding is consistent with previous results from the FAMIPASS studies, which revealed a more significant role for mothers than fathers in shaping their children’s PA and sleep patterns [16, 17]. An explanation for the more prominent role of mothers than fathers in instilling health-promoting 24-hMB patterns in their offspring may lie in parenting styles - mothers tend to be more permissive, responsive, and supportive, while fathers tend to be more restrictive, coercive, and harsher, showing less parental concern than mothers [44]. Another possible reason for the more prominent role of mothers than fathers in child rearing could be the relatively high divorce rate in Czechia. Although significantly reduced from the previous decade, the divorce rate was still 45% in 2021, with 61% of those divorces involving minor children [45]. Subsequently, in 80% of cases, the minor child was placed in the custody of the mother, in 12% of cases, both parents had alternate or joint custody, and in the remaining cases, the child was placed in the custody of the father or another person [46].

### Practical applications

For parents, the simplest daily tool to promote children’s adherence to 24-hMBGS is walking outside with them for at least 25 min a day (or once every two days for at least 50 min), allowing even children who do not participate in OPA to reach the MVPA level of those actively involved in OPA. In addition, we encourage parents to motivate their children to participate in structured leisure activities with a physical, artistic, nature-care, spiritual, or other focus, incorporating active transportation (walking, cycling, skateboarding or using scooters) to and from these activities. Given the workload of parents, it is understandable that there is little time for family PA on work/school days, which can be compensated by regular joint outdoor trips and PA on weekend days.

### Strengths and limitations of the study

A distinct strength of this study was the accelerometer-based continuous 24-hMB monitoring of a representative sample of Czech children aged 3–10 years attending a regular kindergarten/school class on a daily basis. A potential limitation of the research was voluntary nature and personal interest of participants, as it can be reasonably assumed that families with intrinsic motivation to participate free of charge in the demanding weekly monitoring of exercise regimen may tend to adopt a healthy lifestyle more than families with no interest in participating in the research. We attempted to eliminate this limitation by emphasizing the non-competitive nature of the research in order to capture the normal movement behaviour regimen involving kindergarten/school instruction. We also did not make specific recommendations to any of the participants about daily sleep time, PA, and sedentary behaviour. Another limitation of the study may have been the failure to ascertain family type and parental marital status (e.g., complete, incomplete, divorced, widowed, children in alternate care, etc.), as we did not want to reduce participant response rates by requesting this sensitive information. In part, marital status may serve as a proxy for surveyed SES in terms of economic power, but this proved to be insignificant in determining the role of children’s active participation in OPA or adherence to the 24-hMBGS. Although follow-up data collection is planned for the same group of participants, the cross-sectional design of this study involving only the baseline stage of data collection precludes inferring causal relationships.

## Conclusions

- Active 3–10-year-old participants in OPA compared to non-participants were significantly more likely to meet at least two of the 24-hMBG (77.7% vs. 66.4%, *p* = 0.008), had significantly shorter daily sedentary time (by 30 min per day, *p* = 0.001) and had a significantly lower prevalence of excessive body weight (10.26% vs. 24.87% *p* < 0.001).
- Active participation in OPA significantly (*p* < 0.01) helped 3–10-year-old children achieve at least two of the 24-hMBG, regardless of their gender, age, excess body weight, and family SES.
- Mothers played a more important role in their children’s compliance with 24-hMBG than fathers. Mother’s non-excessive body weight and attainment of at least two of the 24-hMBG significantly (*p* < 0.05) assisted children in meeting at least two of the 24-hMBG.
- Regular and long-term implementation of at least 80 min of OPA per week, with parental support, appears to be an appropriate lifestyle strategy for achieving at least two of the three 24-hMBG and for preventing the development of excess body weight in young children.

## Data Availability

No datasets were generated or analysed during the current study.

## Abbreviations

BMI: Body mass index
CI: Confidence interval
FAMIPASS: Family physical activity, sedentary behaviour and sleep study
FAS: Family affluence scale
MVPA: Moderate-to-vigorous physical activity
OPA: Organised physical activity
OR: Odds ratio
PA: Physical activity
Ref.: Reference group
SB: Sedentary behaviour
SES: Socioeconomic status
SD: Standard deviation
ST: Screen time
TV: Television viewing
WHO: World health organization
24-hMB: 24-hour movement behaviour
24-hMBGS: 24-hour movement behaviour guidelines
χ^2^: Pearson Chi-square test
%: Percentages

## References

[1] Rollo S, Antsygina O, Tremblay MS. The whole day matters: Understanding 24-hour movement guideline adherence and relationships with health indicators across the lifespan. J Sport Health Sci. 2020;9:493–510. 10.1016/j.jshs.2020.07.004 32711156 10.1016/j.jshs.2020.07.004 PMC7749249

[2] Rhodes RE, Guerrero MD, Vanderloo LM, Barbeau K, Birken CS, Chaput JP, Faulkner G, Janssen I, Madigan S, Mâsse LC, McHugh TL, Perdew M, Stone K, Shelley J, Spinks N, Tamminen KA, Tomasone JR, Ward H, Welsh F, Tremblay MS. Development of a consensus statement on the role of the family in the physical activity, sedentary, and sleep behaviours of children and youth. Int J Behav Nutr Phys Act. 2020;17(1):74. 10.1186/s12966-020-00973-0 32539730 10.1186/s12966-020-00973-0 PMC7296673

[3] Gustafson SL, Rhodes R. Parental correlates of physical activity in children and early adolescents. Sports Med. 2006;36(1):79–97. 16445312 10.2165/00007256-200636010-00006

[4] Goncalves WSF, Byrne R, de Lira PIC, Viana MT, Trost SG. Adherence to 24-hour movement guidelines among rural Brazalian preschool children: associations with parenting practices. Int J Behav Nutr Phys Act. 2022;19:133. 10.1186/s12966-022-01369-y 36271449 10.1186/s12966-022-01369-y PMC9587598

[5] Fridolfsson J, Buck C, Hunsberger M, Baran J, Lauria F, Molnar D, Moreno LA, Börjesson M, Lissner L, Arvidsson D. I.Family consortium. High-intensity activity is more strongly associated with metabolic health in children compared to sedentary time: a cross-sectional study of the i.family cohort. Int J Behav Nutr Phys Act. 2021;18(1):90. 10.1186/s12966-021-01156-1 34229708 10.1186/s12966-021-01156-1 PMC8261968

[6] Herbert JJ, Møller NC, Andersen LB, Wedderkopp N. Organized sport participation is associated with higher levels of overall health-related physical activity in children (CHAMPS Study-DK). PLoS ONE. 2015;10(8):e0134621. 10.1371/journal.pone.0134621 26262678 10.1371/journal.pone.0134621 PMC4532417

[7] Rocha HA, Marinho DA, Jidovtseff B, Silva AJ, Costa AM. Influence of regular soccer or swimming practice on gross motor development in childhood. Motricidade. 2016;12(4):33–43. 10.6063/motricidade.7477

[8] Chowdhury J. Effect of sport participation on social development in children ages 6–14. CHILD J. 2023;2(1):child.v2i1.3474. 10.15173/child.v2i1.3474.

[9] Harlow M, Wolman L, Fraser-Thomas J. Should toddlers and preschoolers participate in organized sport? A scoping review of developmental outcomes associated with young children’s sport participation. Int Rev Sport Exerc Psychol. 2018;13(1):40–64. 10.1080/1750984X.2018.1550796

[10] Taylor RW, Haszard JJ, Healey D, Meredith-Jones KA, Taylor BJ, Galland BC. Adherence to 24-h movement behavior guidelines and psychosocial functioning in young children: a longitudinal analysis. Int J Behav Nutr Phys Act. 2021;18(1):110. 10.1186/s12966-021-01185-w 34433476 10.1186/s12966-021-01185-w PMC8385859

[11] Kuzik N, Spence JC, Arkko K, Blye CJ, Davie J, Duddridge R, Ekeli T, English A, Etruw E, Hunter S, Lamboglia CG, Nesdoly A, Predy M, Rubuliak R, Wohlers B, Wright K, Carson V. Associations between meeting the Canadian 24-hour movement guidelines and physical, cognitive, social-emotional, and overall development in early childhood.J Act Seden Sleep Behav. 2022;1:2(2022). 10.1186/s44167-022-00002-4 10.1186/s44167-022-00002-4 PMC11934447 40229982

[12] Bjørnarå HB, Westergren T, Sejersted E, Torstveit MK, Hansen BH, Berntsen S, Bere E. Does organized sports participation in childhood and adolescence positively influence health? A review of reviews. Prev Med Rep. 2021;23:101425. 10.1016/j.pmedr.2021.101425 34150481 10.1016/j.pmedr.2021.101425 PMC8190469

[13] Sigmundová D, Dygrýn J, Vorlíček M, Banátová K, Voráčová J, Sigmund E. FAMIly physical activity, sedentary behaviour and sleep (FAMIPASS) study: protocol for a cross-sectional study. BMJ Open. 2023;13(8):e073244. 10.1136/bmjopen-2023-073244 37550023 10.1136/bmjopen-2023-073244 PMC10407347

[14] Sigmund E, Sigmundová D, Badura P, Voráčová J, Vladimír H Jr, Hollein T, Pavelka J, Půžová Z, Kalman M. Time-trends and correlates of obesity in Czech adolescents in relation to family socioeconomic status over a 16-year study period (2002–2018). BMC Pub Health. 2020;20(1):229. 10.1186/s12889-020-8336-2 32054463 10.1186/s12889-020-8336-2 PMC7020383

[15] Sigmund E, Sigmundová D, Pavelka J, Kalman M, Voráčová J, Meier Z, Kopčáková J, Badura P. Changes in the prevalence of obesity in Czech adolescents between 2018 and 2022 and its current non-genetic correlates - HBSC study. BMC Pub Health. 2023;23(1):2092. 10.1186/s12889-023-17010-x 37880679 10.1186/s12889-023-17010-x PMC10601351

[16] Sigmundová D, Voráčová J, Dygrýn J, Vorlíček M, Sigmund E. Parent-Child associations in Accelerometer-Measured physical activity and sedentary behaviour: the FAMIPASS study. Child (Basel). 2024;11(6):710. 10.3390/children11060710 10.3390/children11060710 PMC11202232 38929289

[17] Voráčová J, Sigmund E, Vorlíček M, Dygrýn J, Sigmundová D. Accelerometer-measured sleep behaviour and parent-child sleep guideline adherence and sleep quality in Czech families with children aged 3–8 years: the family physical activity, sedentary behaviour and sleep (FAMIPASS) study. J Sleep Res. 2024;33(6):e14242. 10.1111/jsr.14242 38757216 10.1111/jsr.14242 PMC11597020

[18] Sigmund E, Voráčová J, Dygrýn J, Vorlíček M, Sigmundová D. Comparative analysis of 24-h movement behaviours in Non-Overweight and overweight/obese children: findings from the family physical activity, sedentary behaviour, and sleep (FAMIPASS). Child (Basel). 2024;11(11):1298. 10.3390/children11111298 10.3390/children11111298 PMC11592573 39594873

[19] Migueles JH, Rowlands AV, Huber F, Sabia S. GGIR: A research community–driven open source R package for generating physical activity and sleep outcomes from multi-day Raw accelerometer data. J Meas Phys Behav. 2019;2:188–96. 10.1123/jmpb.2018-0063

[20] van Hees VT, Sabia S, Jones SE, Wood AR, Anderson KN, Kivimäki M, Frayling TM, Pack AI, Bucan M, Trenell MI, Mazzotti DR, Gehrman PR, Singh-Manoux BA, Weedon MN. Estimating sleep parameters using an accelerometer without sleep diary. Sci Rep. 2018;8:12975. 10.1038/s41598-018-31266-z 30154500 10.1038/s41598-018-31266-z PMC6113241

[21] Zborilova V, Pridalova M, Sigmundova D, Kaplanova T. The validity of parental-reported body height and weight: a comparison with objective measurements of 7-8-year-old Czech children. Anthrop Rev. 2018;81(3):278–88. 10.2478/anre-2018-0027

[22] Chai LK, Collins CE, May C, Holder C, Burrows TL. Accuracy of Parent-Reported child height and weight and calculated body mass index compared with objectively measured anthropometrics: secondary analysis of a randomized controlled trial. J Med Internet Res. 2019;21(9):e12532. 10.2196/12532 31538954 10.2196/12532 PMC6754693

[23] Chan NP, Choi KC, Nelson EA, Sung RY, Chan JC, Kong AP. Self-reported body weight and height: an assessment tool for identifying children with overweight/obesity status and cardiometabolic risk factors clustering. Matern Child Health J. 2013;17(2):282–91. 10.1007/s10995-012-0972-4 22395818 10.1007/s10995-012-0972-4

[24] Torsheim T, Cavallo F, Levin KA, Schnohr C, Mazur J, Niclasen B, Currie C, FAS Development Study Group. Psychometric validation of the revised family affluence scale: a latent variable approach. Child Indic Res. 2016;9:771–84. 10.1007/s12187-015-9339-x 27489572 10.1007/s12187-015-9339-x PMC4958120

[25] Hobza V, Hamrik Z, Bucksch J, De Clerq B. The family affluence scale as an indicator for socioeconomic status: validation on regional income differences in the Czech Republic. Int J Environ Res Public Health. 2017;14(12):E1540. 10.3390/ijerph14121540 PMC5750958 29292773

[26] Schmitz KH, Harnack L, Fulton JE, Jacobs DR Jr, Gao S, Lytle LA, Van Coevering P. Reliability and validity of a brief questionnaire to assess television viewing and computer use by middle school children. J Sch Health. 2004;74(9):370–7. 10.1111/j.1746-1561.2004.tb06632.x 15656264 10.1111/j.1746-1561.2004.tb06632.x

[27] WHO. Guidelines on physical activity, sedentary behaviour and sleep for children under 5 years of age. Geneva: World Health Organization. 2019 https://www.who.int/publications/i/item/9789241550536 (Accessed 4 Dec 2024). 31091057

[28] WHO. WHO guidelines on physical activity and sedentary behaviour. Geneva: World Health Organization. 2020. https://www.who.int/publications/i/item/9789240015128 (Accessed 4 Dec 2024).

[29] Tremblay MS, Carson V, Chaput JP, Connor Gorber S, Dinh T, Duggan M, Faulkner G, Gray CE, Gruber R, Janson K, Janssen I, Katzmarzyk PT, Kho ME, Latimer-Cheung AE, LeBlanc C, Okely AD, Olds T, Pate RR, Phillips A, Poitras VJ, Rodenburg S, Sampson M, Saunders TJ, Stone JA, Stratton G, Weiss SK, Zehr L. Canadian 24-Hour movement guidelines for children and youth: an integration of physical activity, sedentary behaviour, and sleep. Appl Physiol Nutr Metab. 2016;41(6 Suppl 3):S311–27. 10.1139/apnm-2016-0151 27306437 10.1139/apnm-2016-0151

[30] Ross R, Ross R, Chaput JP, Giangregorio LM, Janssen I, Saunders TJ, Kho ME, Poitras VJ, Tomasone JR, El-Kotob R, McLaughlin EC, Duggan M, Carrier J, Carson V, Chastin SF, Latimer-Cheung AE, Chulak-Bozzer T, Faulkner G, Flood SM, Gazendam MK, Healy GN, Katzmarzyk PT, Kennedy W, Lane KN, Lorbergs A, Maclaren K, Marr S, Powell KE, Rhodes RE, Ross-White A, Welsh F, Willumsen J, Tremblay MS, et al. Canadian 24-Hour movement guidelines for adults aged 18–64 years and adults aged 65 years or older: an integration of physical activity, sedentary behaviour, and sleep. Appl Physiol Nutr Metab. 2020;45(10):S57–102. 10.1139/apnm-2020-0467. (Suppl. 2)). 33054332 10.1139/apnm-2020-0467

[31] WHO Multicentre Growth Reference Study Group, de Onis M. WHO child growth standards based on length/height, weight and age. Acta Pædiatr. 2006;95:76–85. 10.1111/j.1651-2227.2006.tb02378.x 10.1111/j.1651-2227.2006.tb02378.x 16817681

[32] de Onis M, Onyango AW, Borghi E, Siyam A, Nishida C, Siekmann J. Development of a WHO growth reference for school-aged children and adolescents. Bull World Health Organ. 2007;85(9):660–7. 10.2471/blt.07.043497 18026621 10.2471/BLT.07.043497 PMC2636412

[33] Woynarowska B, Palczewska I, Oblacińska A. Standardy who Rozwoju fizycznego Dzieci W Wieku 0–5 Lat. Siatki Centylowe Dlugosci/wysokosci Masy Ciala Wskaznika Masy BMI I Obwodu glowy [WHO child growth standards for children 0–5 years. Percentile charts of length/height, weight, body mass index and head circumference]. Med Wieku Rozwoj. 2012;16(3):232–9. 23378401

[34] World Health Organization. Overweight and Obesity. 2024. Available online: https://www.who.int/news-room/fact-sheets/detail/obesity-and-overweight (accessed on 11 December 2024).

[35] Tapia-Serrano MA, Sevil-Serrano J, Sánchez-Miguel PA, López-Gil JF, Tremblay MS, García-Hermoso A. Prevalence of meeting 24-Hour movement guidelines from pre-school to adolescence: A systematic review and meta-analysis including 387,437 participants and 23 countries. J Sport Health Sci. 2022;11(4):427–37. 10.1016/j.jshs.2022.01.005 35066216 10.1016/j.jshs.2022.01.005 PMC9338333

[36] Chong KH, Suesse T, Cross PL, Ryan ST, Aadland E, Aoko O, Byambaa A, Carson V, Chaput JP, Christian H, Cliff DP, De Craemer M, de Lucena Martins CM, Delisle Nyström C, Draper CE, El Hamdouchi A, Florindo AA, Guan H, Ha AS, Hamzavi Zarghani N, Hesketh KD, Hossain MS, Jajat J, Kim T, Koh D, Kontsevaya AV, Kuzik N, Leppänen MH, Löf M, Lubree H, Meredith-Jones K, Mwase-Vuma TW, Ng JYY, Novotny R, Nusurupia JJ, Pham BN, Poh BK, Reilly JJ, Staiano AE, Sultoni K, Tanaka C, Tang HK, Taylor RW, Tomaz SA, Tremblay MS, Trost SG, Turab A, Vale S, Wickramasinghe VP, Okely AD. Pooled analysis of physical activity, sedentary behavior, and sleep among children from 33 countries. JAMA Pediatr. 2024;178(11):1199–207. 10.1001/jamapediatrics.2024.3330 39348138 10.1001/jamapediatrics.2024.3330 PMC11443432

[37] Kracht CL, Webster EK, Staiano AE. Sociodemographic differences in young children meeting 24-Hour movement guidelines. J Phys Act Health. 2019;16(10):908–15. 10.1123/jpah.2019-0018 31491748 10.1123/jpah.2019-0018 PMC7058481

[38] Byambaa A, Dechinjamts O, Jambaldorj B, Jones RA, Chong KH, Okely AD. Prevalence and health associations of meeting the world health organization guidelines for physical activity, sedentary behavior, and sleep in Preschool-Aged children: the SUNRISE Mongolia pilot and feasibility study. J Phys Act Health. 2024;21(3):283–93. 10.1123/jpah.2023-0511 38242111 10.1123/jpah.2023-0511

[39] Coyle-Asbil HJ, Breau B, Ma DWL, Haines J, Buchholz AC, Vallis LA. Guelph family health study. Compliance with the 24-hour movement behavior guidelines and the impact of sleep methods among toddler, preschool, and school-aged children enrolled in the Guelph family health study. J Sci Med Sport. 2024;27(9):631–39. 10.1016/j.jsams.2024.05.014 38937183 10.1016/j.jsams.2024.05.014

[40] Swindell N, Wachira LJ, Okoth V, Kagunda S, Owino G, Ochola S, Brophy S, Summers H, Richards A, Fairclough SJ, Onywera V, Stratton G. Prevalence and correlates of compliance with 24-h movement guidelines among children from urban and rural Kenya-The Kenya-LINX project. PLoS ONE. 2022;17(12):e0279751. 10.1371/journal.pone.0279751 36584149 10.1371/journal.pone.0279751 PMC9803245

[41] Feng J, Huang WY, Reilly JJ, Wong SH. Compliance with the WHO 24-h movement guidelines and associations with body weight status among preschool children in Hong Kong. Appl Physiol Nutr Metab. 2021;46(10):1273–8. 10.1139/apnm-2020-1035 33945770 10.1139/apnm-2020-1035

[42] Leppänen MH, Ray C, Wennman H, Alexandrou C, Sääksjärvi K, Koivusilta L, Erkkola M, Roos E. Compliance with the 24-h movement guidelines and the relationship with anthropometry in Finnish preschoolers: the DAGIS study. BMC Public Health. 2019;19(1):1618. 10.1186/s12889-019-7967-7 31796014 10.1186/s12889-019-7967-7 PMC6889540

[43] De Craemer M, Verbestel V, Cardon G, Androutsos O, Manios Y, Chastin S. Correlates of meeting the physical activity, sedentary behavior, and sleep guidelines for the early years among Belgian preschool children: the ToyBox-Study. Int J Environ Res Public Health. 2020;17(19):7006. 10.3390/ijerph17197006 32987961 10.3390/ijerph17197006 PMC7579535

[44] Yaffe Y. Systematic review of the differences between mothers and fathers in parenting styles and practices. Curr Psychol. 2023;42:16011–24. 10.1007/s12144-020-01014-6

[45] Český Statistický úřad (ČSÚ). Vývoj obyvatelstva v krajích České republiky, Rozvodovost [Population development in the regions of the Czech Republic, Diversity]. https://csu.gov.cz/docs/107508/1cc1988d-ff40-a111-4118-245c4bc918fd/1301572203.pdf?version=1.0 Accessed 16 Dec 2024.

[46] Bold F. Péče o nezletilé děti po rozvodu/rozchodu rodičů [Care of minor children after divorce/separation of parents]. Právní Prostor. 2019; 1–5. https://www.pravniprostor.cz/clanky/obcanske-pravo/pece-o-nezletile-deti-po-rozvodu-rozchodu-rodicu Accessed 16 Dec 2024.

